# Evaluating Medical Student Perceptions of the ADEPT-CARE Protocol Through Remote, Self-Directed Learning

**DOI:** 10.7759/cureus.84041

**Published:** 2025-05-13

**Authors:** Carly Malesky, Sandra Carpenter, Heather McClure, Weston Carpenter, Rong Wu, Zita Lazzarini

**Affiliations:** 1 School of Medicine, University of Connecticut School of Medicine, Farmington, USA; 2 General Surgery, Beth Israel Deaconess Medical Center, Harvard Medical School, Boston, USA; 3 Biostatistics, The Cato T. Laurencin Institute for Regenerative Engineering, University of Connecticut School of Medicine, Farmington, USA; 4 Public Health Sciences, University of Connecticut School of Medicine, Farmington, USA

**Keywords:** curriculum development and evaluation, disability, inclusivity, medical education curriculum, remote assessment

## Abstract

Background: Individuals with disabilities represent the largest minority group in the United States, yet they face significant barriers to accessing healthcare, resulting in notable health disparities. Despite the high prevalence of disabilities in patient populations, standardized disability training is often absent from undergraduate medical curricula. The “ADEPT-CARE” protocol is an educational framework developed to enhance the ability of medical trainees to interact effectively with disabled patients. Prior pilot studies have shown that the ADEPT-CARE protocol, included in an elective course for pre-clinical medical students, improves their perceptions and confidence in caring for disabled patients.

Methods: From April 2022 to September 2023, medical students at the University of Connecticut School of Medicine participated in the ADEPT-CARE training. Participants, including students from all years of study, completed a pre-module survey, watched an ADEPT-CARE teaching video, and completed a post-module survey within 24 hours. The training was conducted remotely and was self-directed. Statistical analysis of the pre- and post-survey responses was performed using nonparametric Wilcoxon signed-rank tests to assess changes in student perceptions and confidence.

Results: A total of 40 students completed the training. Among them, 12.5% identified as having a disability, while 50% reported having a family member with a disability. Post-training, students demonstrated a statistically significant increase in confidence regarding the assessment of patients with disabilities (p = 0.0005) and were more likely to employ a consistent approach during patient encounters (p < 0.001). Additionally, students felt more capable of providing equitable care to patients with disabilities (p = 0.0002).

Conclusions: The study indicates that self-directed, remote exposure to the ADEPT-CARE protocol can enhance medical students’ confidence in treating patients with disabilities. Future research should explore the protocol’s effectiveness in clinical settings and its feasibility during simulated patient encounters. The ADEPT-CARE protocol proves to be an effective method for integrating disability-focused training into undergraduate medical education.

## Introduction

As of 2022, more than 25% of adults in the United States (U.S.) identify as living with a disability, and this percentage has been persistently rising [1]. Although patients with disabilities are frequent consumers of healthcare, they regularly encounter significant barriers to their care stemming from inaccessible healthcare environments and programs. The current lack of disability-specific training for physicians at all levels of medical education contributes to these health disparities [2,3]. Surveys of practicing physicians found that less than half feel confident they could provide an equal quality of care to disabled patients [3]. Furthermore, studies demonstrate that many physicians are unfamiliar with legal protections afforded to disabled patients, such as the right to reasonable accommodation, and hold negative attitudes toward their patients with disabilities [3]. For example, the majority of surveyed practicing physicians believe that patients with disabilities have a worse quality of life compared to nondisabled patients, even though disabled patients consistently rate their quality of life the same as their able-bodied counterparts [3].

The state of disability training in U.S. medical schools is inadequate. Disability education is often omitted from pre-clinical didactics, resulting in students feeling unprepared to care for disabled individuals when they begin clinical clerkships [4]. Based on limited surveys of medical school deans, it is likely that less than half of U.S. medical schools provide any disability-focused training [2]. This training is highly variable between schools. Moreover, the format of training is often limited to panels in a large-group setting designed to promote “disability awareness,” but it is inadequate in showing a range of disabilities and teaching clinically applicable skills [2]. Surveys indicate that 80% of students exposed to this type of instruction believe it does not prepare them to interact with disabled patients in day-to-day practice [5].

There is currently a need for standardized, longitudinal training on disability that can be flexibly incorporated into both pre-clinical and clinical education [3,4]. In a systematic review of 63 studies, healthcare barriers for individuals with disabilities were categorized into six different groups: training, knowledge, communication skills, fear, time, and involvement in decision-making [5]. The ADEPT-CARE protocol addresses all of these barriers and aims to improve physician-patient communication, cultural dexterity, and examination skills for patients with a range of disability. In 2022, ADEPT-CARE was evaluated at the Penn State College of Medicine as a pilot study. The protocol was integrated into an elective course on disability for first-year medical students [6]. The results of the pilot study showed that 95% of students reported they understood the mnemonic and that they planned to use it in future clinical practice [6]. In addition, 86% of these students agreed that ADEPT-CARE provided them with a consistent method for assessing an individual with a disability [6]. Limitations of the pilot study included selection bias and generalizability, as the ADEPT-CARE protocol was integrated into an elective disability course for first-year students. The objective of this study was to assess the efficacy of the ADEPT-CARE protocol as a remote, stand-alone intervention among medical students of all levels. Specifically, we aimed to evaluate its impact on students' confidence in providing equitable care to patients with disabilities and their intent to apply a consistent approach for assessing these patients in future clinical practice.

## Materials and methods

Medical students of all levels (first to fourth year) at the University of Connecticut School of Medicine were eligible to participate in this study under Institutional Review Board approval (#22X-269-2). From April 2022 to September 2023, students were contacted via recruitment emails to voluntarily and remotely complete a pre-module survey (5-10 minutes) and then watch the ADEPT-CARE teaching video (20-30 minutes). Following the video, students were then asked to complete a post-module survey (5-10 minutes) within a 24-hour period. Pre- and post-surveys were matched by a participant-generated unique anonymous survey identification code. All survey responses were measured using Likert-type scales. Scales 1 to 4 were used in four-item responses, such as “strongly agree,” “somewhat agree,” “somewhat disagree,” and “strongly disagree.” Scales 1 to 4 also corresponded to “very confident,” “somewhat confident,” “not very confident,” and “not at all confident.” Similarly, scales 1 to 5 corresponded to the five-item response, i.e., “a lot better,” “a little better,” “the same,” “a little worse,” and “a lot worse.” A low-value incentive ($50 gift card raffle) was provided for participation. This was implemented halfway through the study period to encourage participation from clinical medical students.

Data was reviewed at two different time points by the same researcher to ensure consistency and accuracy. General characteristics of the sample were described utilizing descriptive statistics. Pre-module and post-module scores were compared using nonparametric Wilcoxon signed rank tests. A p-value of <0.05 determined statistical significance. The sample size was estimated using the asymptotic relative efficiency (ARE) method. Based on a minimum ARE of 0.864, a sample size of 40 for the Wilcoxon signed-rank test was considered equivalent to a sample size of 35 for the paired t-test, providing 82% power to detect a pre-post difference of 0.4, assuming a standard deviation of 0.8 and a two-sided alpha level of 0.05. To account for an anticipated 20% attrition rate, we initially planned to recruit 50 participants. The final analytic sample included 40 matched surveys, after excluding nine entries due to incomplete data or duplicate participation. In cases of duplicate responses, the first submission was used for primary analysis, while the second was referenced to fill in any missing responses from the first. All statistical analyses were performed using SAS 9.4 software (2023; SAS Institute Inc., Cary, North Carolina, United States).

## Results

A total of 40 matched surveys were completed by medical students enrolled at the institution, representing participation from about 10% of the medical student body (40/400 medical students). Of those who completed the pre-survey, 25% identified as male, 72.5% identified as female, and 2.5% identified as non-binary/third gender. The majority of participants (55%) were between the ages of 20-24, followed by 42.5% aged 25-29, and 2.5% aged 30-40. A total of 12.5% of pre-survey participants identified as a person with a disability, and 50% of participants reported having someone close to them in their life who identifies as a person with a disability. The majority of participants were first-year medical students (40%), followed by second-year medical students (25%). Third- and fourth-year medical students each represented 17.5% of total respondents (Table 1).

**Table 1:** Demographic characteristics of the study population ADA: The Americans with Disabilities Act (A) Participant who chose to self-describe responded “Arab.” (B) Per the ADA, a person with a disability is someone who “has a physical or mental impairment that substantially limits one or more major life activities,” “has a history or record of such an impairment,” or “is perceived by others as having such an impairment”

	N	%
Gender identity		
Female	29	25
Male	10	72.5
Non-binary/third gender	1	2.5
Age (years)		
20-24	22	55
25-29	17	42.5
30-40	1	2.5
Race/ethnicity		
Asian	5	12.5
Black or African American	5	12.5
Hispanic	2	5
White	24	60
Other	1	2.5
Self-Describe^ A^	1	2.5
Prefer not to say	2	5
Year in training		
M1	16	40
M2	10	25
M3	5	12.5
M4	7	17.5
Research year or MD/PhD	2	5
Disability identity^ B^		
Yes	5	12.5
No	34	85
Prefer not to say	1	2.5

A total of 71.8% of participants reported previous experience working with individuals with disabilities. Moreover, 35.9% of participants indicated having formal training on disability at some point in their education, and 41.0% of participants had no formal training. In the average month, a majority of respondents (61.5%) reported that it was rare that they took care of patients with a disability.

Following exposure to the ADEPT-CARE protocol, students reported greater confidence in their ability to assess patients with a disability (p = 0.0005), and they stated that they were more likely to utilize a consistent approach for assessment (p < 0.001). Students reported increased confidence in their ability to provide equitable care to patients with disabilities (p = 0.0002) and believed that the quality of life for disabled patients improved after exposure to the ADEPT-CARE protocol (p = 0.047). There was no difference in student perception of treatment of patients with disabilities by the healthcare system or need for additional training to care for this patient population (p > 0.5). A majority of students agreed both pre- and post-intervention that more training was needed to care for patients with disabilities, a perception that did not change after viewing the ADEPT-CARE protocol (Table 2).

**Table 2:** ADEPT-CARE survey results *Statistically significant at p < 0.05 Data is presented as a percentage of combined agree/strongly agree. Analyses were performed using a raw four-point Likert scale

Statement	Pre-survey	Post-survey	Wilcoxon signed-rank test p-value
	N (%) agree/strongly agree or confident/very confident		
Working with people with disabilities will be important for my specialty or intended specialty	39 (97.5)	40 (100)	0.56
I would welcome patients with disabilities into my practice	38 (100)	39 (100)	1.0
People with disabilities are often treated unfairly in the healthcare system	38 (100)	39 (94.88)	0.79
The treatment of patients with disabilities is too time-consuming	5 (13.16)	4 (10.25)	0.56
Understanding my patients with disabilities is valuable to me as a physician	38 (100)	39 (100)	0.25
In general, compared with people without disabilities, do you believe the overall quality of life of people with significant disabilities is...			0.047*
A lot better	1 (2.63)	0 (0)	
A little better	0 (0)	0 (0)	
The same	9 (23.68)	18 (46.15)	
A little worse	22 (57.89)	16 (41.03)	
A lot worse	6 (15.79)	5 (12.82)	
How confident are you in your ability to assess a patient with a disability	26 (66.67)	37 (92.5)	0.0005*
How confident are you in your ability to provide the same quality of care to patients with disabilities as you provide to patients without disabilities	26 (66.67)	37 (92.5)	0.0002*
More training is needed to prepare physicians to provide care to patients with disabilities	39 (100)	40 (100)	0.73
People with disabilities experience barriers to healthcare that physicians can help to address	39 (100)	40 (100)	0.69
When I am assessing a patient with a disability, I have a consistent approach or strategy in mind	12 (30.77)	30 (75)	<0.0001*

In both pre- and post-surveys, students were more likely to agree that there are additional barriers that patients with disabilities face in a healthcare setting and that patients with disabilities are often unfairly treated by the healthcare system. All but one participant agreed or strongly agreed that working with individuals with disabilities will be important for their specialty or intended specialty post-test. Additionally, all participants agreed or strongly agreed that understanding patients with disabilities is valuable for physicians, and this did not change after exposure to ADEPT-CARE. All participants also agreed or strongly agreed that more training is needed to prepare physicians to care for patients with disabilities. Furthermore, all participants agreed or strongly agreed that they would welcome patients with disabilities into their practice. Regarding the statement "treatment of patients with disabilities is too time-consuming," there was a shift in responses from pre- to post-survey, with a greater number of participants strongly disagreeing, but the change was not statistically significant.

## Discussion

The results of this study demonstrate that the ADEPT-CARE protocol increases student confidence in both their assessment and care of patients with disabilities. In contrast to previous studies on the ADEPT-CARE protocol, which showed no change in confidence among participants in assessing patients with disabilities, respondents in this study reported feeling more confident after their exposure to the protocol [6]. Our study population was relatively inexperienced in caring for patients with disabilities. Half the respondents reported having someone close to them who identifies as having a disability, but over half had not clinically taken care of a patient with a disability. Although participants had personal relationships and experiences with individuals with disabilities, this prior exposure does not automatically translate to better clinical practice.

ADEPT-CARE is a flexible, standardized teaching tool for healthcare professionals to use in their care of patients with disabilities. It can be incorporated in a remote and self-directed manner, which may be helpful for older students who are in clinical clerkships and no longer have large-group didactics. Exposure to ADEPT-CARE as a stand-alone intervention has been shown to change student perceptions and provide learners with a disability-specific framework for patient care.

The protocol has now demonstrated efficacy in at least two different medical schools [6,7]. Importantly, our study contributes to the evaluation of ADEPT-CARE by administering it as an isolated intervention and not part of any disability-focused course. As medical education continues to evolve, integrating innovative, self-directed learning tools, such as spaced repetition platforms, can supplement protocols like ADEPT-CARE to reinforce long-term retention and competency across specialties [8]. This study also provides a larger sample size than the pilot study after pre- and post-survey matching. Furthermore, this study demonstrated a statistically significant improvement in students’ reported confidence in caring for patients with disabilities, unlike the prior pilot study [6]. The findings at our institution support the reproducibility of the ADEPT-CARE protocol as an asynchronous and feasible teaching tool for disability-focused medical curricula.

Our study has several limitations. One important limitation was the overall low participation rate, especially for clinical medical students. The surveys and teaching modules required a total of approximately 45 minutes to complete, which may have been a deterrent for clinical medical students who have less extracurricular time. As the intervention was voluntary and not associated with any elective or mandatory course, students were less likely to participate, even in spite of offering an incentive halfway through the study and extension of the study period. Perceptions of disability are likely to change throughout medical training as students encounter patients with disabilities and develop attitudes toward disability that may be beneficial or harmful. Students earlier in their training with less exposure to disability in healthcare settings may be more comparable to one another and with attitudes more amenable to change through didactics. Another important limitation is selection bias. 8.3% of medical students anonymously self-reported a disability on the Association of American Medical Colleges (AAMC) graduation questionnaire in 2021 [1]. In the pilot study at Penn State, only 3% of participants self-reported having a disability. However, in our study, 12.5% of respondents self-reported having a disability, which is slightly higher than both the AAMC graduation questionnaire and pilot study conducted at Penn State. However, having a self-reported disability does not necessarily mean that they have had disability-specific medical training, and changes in attitudes may still be significant. Variability in disability status could be attributed to the active disabilities interest group at the school of medicine, making them more inclined to take the survey due to interest in its content, leading to more favorable opinions about patients with disabilities. Data collection was conducted through unsupervised self-report questionnaires, which may introduce biases related to misunderstanding or misreporting of items. This study did not include objective validation of self-reported data, which may be subject to recall bias or social desirability bias.

The benefit of ADEPT-CARE is that it can be flexibly incorporated because of its broad applicability and incorporation of a range of disabilities. The implementation of ADEPT-CARE across various settings at different time points will promote sustained and longitudinal utilization of the framework. In the future, we aim to assess the efficacy of the ADEPT-CARE protocol in clinical settings during simulated patient encounters and in real-life patient interactions on clinical clerkships. Results from this study support the effectiveness of the protocol on influencing students’ perceptions of disability and intention to use ADEPT-CARE as a consistent framework for interacting with disabled patients. Through exposure of students and physicians at all levels in their training, we hope to more broadly improve the healthcare system’s attitudes and confidence when working with patients who have disabilities. We also plan to investigate the barriers to implementing this protocol clinically and to survey patients on their experience of a visit being conducted using this framework.

## Conclusions

With the rising prevalence of disabilities and the substantial barriers that people with disabilities encounter, adequate training of healthcare professionals to provide patient-centered care for this population is imperative. These findings indicate that the ADEPT-CARE protocol effectively increases medical students’ reported confidence in caring for patients with disabilities. Implementation of this simple educational tool strives to address the well-studied barriers to equitable care for the disabled patient population. We anticipate that incorporating the ADEPT-CARE protocol into the medical school curricula will help trainees prepare for clinical encounters with disabled patients. Further research is needed to examine the efficacy of ADEPT-CARE among medical trainees in clinical settings, including both simulated and direct patient encounters, as well as to better understand the patient experience while using this framework.

## References

[1] (2025). Association of American Medical Colleges (2021) medical school graduation questionnaire: all schools summary report. https://www.aamc.org/data-reports/students-residents/report/graduation-questionnaire-gq.

[2] (2025). CDC data shows over 70 million U.S. adults reported having a disability. https://www.cdc.gov/media/releases/2024/s0716-Adult-disability.html.

[3] Chardavoyne PC, Henry AM, Sprow Forté K (2022). Understanding medical students’ attitudes towards and experiences with persons with disabilities and disability education. Disabil Health J.

[4] Doherty AJ, Atherton H, Boland P (2020). Barriers and facilitators to primary health care for people with intellectual disabilities and/or autism: an integrative review. BJGP Open.

[5] Iezzoni LI, Rao SR, Ressalam J (2021). Physicians' perceptions of people with disability and their health care. Health Aff (Millwood).

[6] Smeltz L, Carpenter S, Benedetto L (2023). ADEPT-CARE: a pilot, student-led initiative to improve care for persons with disabilities via a novel teaching tool. Disabil Health J.

[7] Smeltz L, Carpenter S, Benedetto L (2024). Introduction to disability and antiableist health care: a pilot, student-led module for preclinical medical students. Am J Phys Med Rehabil.

[8] Koenig ZA, Henderson JT, Brooke SM (2022). Creating a spaced repetition model to supplement education in plastic surgery. Plast Reconstr Surg Glob Open.

